# The Mitochondrial Genome Is a “Genetic Sanctuary” during the Oncogenic Process

**DOI:** 10.1371/journal.pone.0023327

**Published:** 2011-08-17

**Authors:** Marcos Seoane, Ana Mosquera-Miguel, Teresa Gonzalez, Maximo Fraga, Antonio Salas, Jose A. Costoya

**Affiliations:** 1 Molecular Oncology Laboratory MOL, Facultade de Medicina, Departamento de Fisioloxia, Universidade de Santiago de Compostela, Galicia, Spain; 2 Unidade de Xenetica, Instituto de Medicina Legal, Facultade de Medicina, Universidade de Santiago de Compostela, Galicia, Spain; 3 Fundacion Galega de Medicina Xenomica, Servicio Galego de Saude, Santiago de Compostela, Galicia, Spain; 4 Departamento de Anatomia Patoloxica e Ciencias Forenses, Universidade de Santiago de Compostela, Galicia, Spain; Thomas Jefferson University, United States of America

## Abstract

Since Otto Warburg linked mitochondrial physiology and oncogenesis in the 1930s, a number of studies have focused on the analysis of the genetic basis for the presence of aerobic glycolysis in cancer cells. However, little or no evidence exists today to indicate that mtDNA mutations are directly responsible for the initiation of tumor onset. Based on a model of gliomagenesis in the mouse, we aimed to explore whether or not mtDNA mutations are associated with the initiation of tumor formation, maintenance and aggressiveness. We reproduced the different molecular events that lead from tumor initiation to progression in the mouse glioma. In human gliomas, most of the genetic alterations that have been previously identified result in the aberrant activation of different signaling pathways and deregulation of the cell cycle. Our data indicates that mitochondrial dysfunction is associated with reactive oxygen species (ROS) generation, leading to increased nuclear DNA (nDNA) mutagenesis, but maintaining the integrity of the mitochondrial genome. In addition, mutational stability has been observed in entire mtDNA of human gliomas; this is in full agreement with the results obtained in the cancer mouse model. We use this model as a paradigm of oncogenic transformation due to the fact that mutations commonly found in gliomas appear to be the most common molecular alterations leading to tumor development in most types of human cancer. Our results indicate that the mtDNA genome is kept by the cell as a “genetic sanctuary” during tumor development in the mouse and humans. This is compatible with the hypothesis that the mtDNA molecule plays an essential role in the control of the cellular adaptive survival response to tumor-induced oxidative stress. The integrity of mtDNA seems to be a necessary element for responding to the increased ROS production associated with the oncogenic process.

## Introduction

Mitochondria are central to cell metabolism, being the principal energy source of the cell, thanks to the cytochrome enzymes of terminal electron transport and the enzymes of the citric acid cycle, fatty acid oxidation, and oxidative phosphorylation. This energy is gradually converted into a proton gradient. Mitochondria use this gradient to synthesize ATP, which is later employed for biosynthetic reactions [1], [2]. On the other hand, changes in mitochondrial membrane permeability lead to the release of proapoptotic mediators regulating a number of signaling cascades, including apoptosis [3], [4]. Therefore, mitochondria are central organelles that control life and death of the cell.

It has been revealed that mitochondrial dysfunction is one of the most common and consistent phenotypes of cancer cells [5]. A number of notable differences in the mitochondria of normal and cancer cells have been described. These include differences in mitochondrial metabolic activity [6], [7], the molecular composition of the mitochondria and the mitochondrial DNA (mtDNA) sequence [8], [9], as well as alterations of nuclear genes that may affect mitochondrial function [10]–[12].

Recent studies have suggested that alterations in mtDNA may be associated with malignant progression, increasing the metastatic potential of tumor cells, and that this phenomenon is mediated by the overproduction of reactive oxygen species [13], [14]. The term “reactive oxygen species” (ROS) encompasses a wide range of molecules. The unpaired electrons of oxygen react to form partially reduced highly reactive species that are classified as ROS, including superoxide radical anions (O_2_^−^) and hydrogen peroxide (H_2_O_2_). Various enzyme systems produce ROS, although mitochondrial oxygen metabolism is the dominant source of ROS and results from the incomplete coupling of electrons and H^+^ with oxygen in the electron transport chain.

Reactive oxygen species are emerging as critical signaling molecules. Traditionally, ROS have been considered as a toxic product of cellular metabolism, but it has become appreciated that they are actively involved in oncogenic signaling in cellular transformation and cancer. Increased numbers of ROS can drive a cycle of genomic instability leading to DNA double-strand breaks (DSBs) and altered repair mechanisms that can result in the acquisition of genomic changes. Increased intracellular levels of ROS have also been reported to mediate some of the biological effects of several oncogenic genes such as HRas^V12^. Among these biological effects, the most important ones are the onset of premature senescence, the generation of genomic instability [15], and malignant transformation [16]. Furthermore, high levels of ROS have been detected in several human cancer cell lines as well as in human tumors from different tissues.

However, it is not only increased levels of intracellular ROS that affect nuclear DNA (nDNA), mtDNA is a particularly vulnerable target because of its proximity to the electron transport chain constituents. Reactive oxygen species mediated mutations in mtDNA have recently emerged as an important variable in carcinogenesis [17]. Moreover, it has been reported that mitochondria play a critical role in replicative senescence and oncogene-induced senescence (OIS), and several mitochondrial changes, including an increase in the production of ROS, were reported in cells with short telomeres [18], [19]. On the other hand, the results of mtDNA instability in tumorigenesis have been questioned under the assumption that most of the apparent findings constituted laboratory or documentation errors [20]–[22].

Taking these reports all together, the present study aims to analyze whether or not there is a real relationship between oxidative stress and mitochondrial alterations in the process of tumorigenesis. In order to carry out these studies, we used a mouse model of gliomagenesis in the hope of understanding the mitochondrial phenotype contributions to the formation of tumors from the beginning of the oncogenic process to high-grade tumors. In addition to the mouse model, entire mtDNA genomes from 11 human glioma patients were sequenced in order to quantify and characterize mtDNA instability.

## Results

### Oncogene-induced ROS generation is associated with chromosomal instability

Previously, in a model of gliomagenesis, we described the resistance of astrocytes to Ras-induced senescence in the presence of high levels of ROS and DNA damage despite DNA damage response (DDR) activation [23]. Moreover, in this context, the loss of the *Rb* locus favors tumor progression and confers a selective advantage to the tumor, increasing the astrocyte's proliferative rate and favoring the inactivation of stress pathways, thus increasing cellular transformation.

To this purpose, conditional Rb mutant mouse astrocytes were infected with a retrovirus-encoding mutant Ras allele (HRas^V12^) and the recombinase Cre that targets the loxP sequences flanking exon 19 of the Rb gene [24]. Thus, we managed to mimic the characteristic upregulation of signaling pathways by introducing a constitutively activated isoform of Ras and cell cycle deregulation by genetically inactivating the pocket protein Rb. In this context, the loss of the *Rb* locus favors the progression of low-grade gliomas to higher-grade tumors. The tumors derived from Ras^V12−^expressing astrocytes bore a strong similarity to human low-grade gliomas, whereas the ones obtained after combining Ras^V12^ expression and Rb loss were similar to high-grade gliomas (glioblastoma multiforme, GBM) [23]. Therefore, in order to examine the role of mtDNA mutations in the development of tumor cells, four groups of primary astrocytes (cRb^loxP/loxP^, Rb^loxP/loxP^/Ras^V12^, cRb^−/−^ and cRb^−/−^/Ras^V12^) and two cell lines derived from tumors formed by primary astrocytes Rb^loxP/loxP^/Ras^V12^ and cRb^−/−^/Ras^V12^ in SCID mice (T653 and T731, respectively) were chosen (Table 1).

**Table 1:** Characterization of cell lines and primary astrocytes.

	Groups	Rb loss	HRas^V12^	Tumor Formation
Primary astrocytes (cRb^loxP/loxP^)	M1/M2	−	−	−
Primary astrocytes (cRb^loxP/loxP^/Ras^V12^)	M3/M4	−	+	+
Primary astrocytes (cRb^−/−^)	M5/M6	+	−	−
Primary astrocytes (cRb^−/−^/Ras^V12^)	M7/M8	+	+	+
T653 cell line	M9	−	+	+
T731 cell line	M10	+	+	+

In the first approach and in order to verify whether or not the expression of oncogenic Ras induces higher levels of intracellular ROS in astrocytes, we measured ROS levels in our primary cultures and cell lines to determine whether or not they differed between the experimental groups.

**Figure 1:**
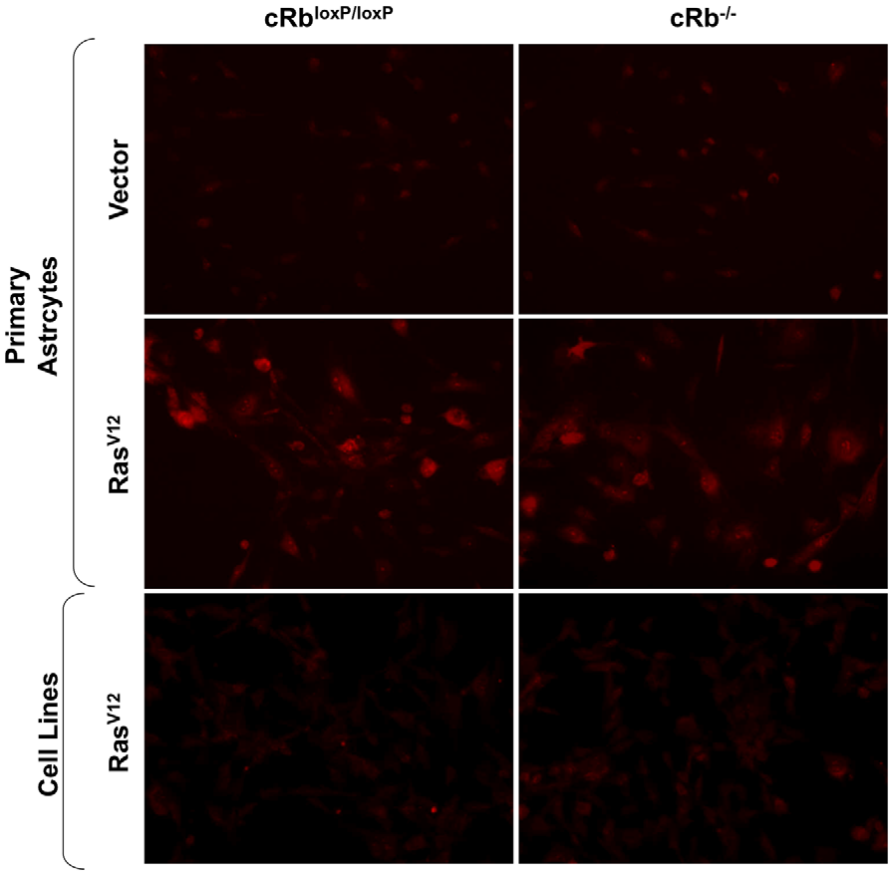
Ras^V12^ expression increases the levels of ROS. Primary astrocytes and cell lines were assayed for dihydroethidium (DHE) fluorescence, indicative of ROS production, and visualized by fluorescent microscopy. Under identical imaging conditions, DHE oxidation was increased in Rb^loxP/loxP^/Ras^V12^ and cRb^−/−^/Ras^V12^ astrocytes.

The HRas^V12^-expressing astrocytes (Rb^loxP/loxP^/Ras^V12^ and cRb^−/−^/Ras^V12^) showed noticeably higher levels of intracellular ROS than cRb^loxP/loxP^ and cRb^−/−^ (Figure 1). As mentioned before, HRas^V12^ has been reported to induce ROS accumulation and this property was linked to the ability of oncogenic Ras to act as a transforming gene [16], [25]. Curiously, the ROS levels detected in cell lines T653 and T731 were lower despite the expression of oncogenic Ras (Figure 1).

Several reports have linked the expression of HRas^V12^ with the induction of DNA damage, chromosomal instability (CIN), and aneuploidy [15], [26], [27]. Moreover, the chromosomal instability caused by HRas^V12^ could be rescued by treating the cells with scavengers of reactive oxygen species [15], [28], [29]. These reports suggest that HRas^V12^ induces genomic instability, at least in part, by increasing intracellular ROS levels [27], [30].

**Figure 2:**
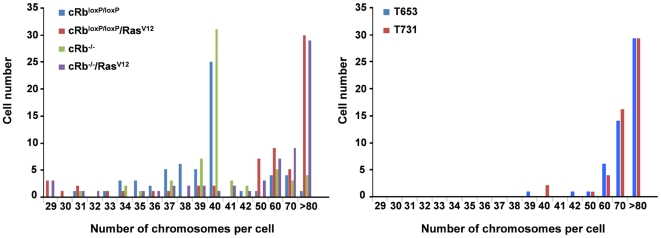
Activated oncogenes: HRas^V12^ promotes chromosomal instability. The primary astrocytes and the cell lines T653 and T731 were treated with colcemid to induce metaphase arrest and then harvested for karyotype analysis. Between 30 and 40 metaphases from three independent infections were counted in each case.

Consistent with these data, we wanted to confirm whether or not the expression of the activated oncogene HRas^V12^ and therefore the high levels of ROS were related to CIN in our astrocytes. To assess the effects of HRas^V12^ and Rb loss, we analyzed the karyotype of the infected primary cultures and cell lines. Both cRb^loxP/loxP^ and cRb^−/−^ primary astrocytes maintained a normal diploid genome (Figure 2). However, a significant proportion of astrocytes became aneuploid in the presence of HRas^V12^ (Rb^loxP/loxP^/Ras^V12^ and cRb^−/−^/Ras^V12^), of which around 30% of these astrocytes presented >80 chromosomes per cell, double the number of chromosomes found in the control cells. Moreover, there was no striking difference in the degree of aneuploidy between Rb^loxP/loxP^/Ras^V12^ and cRb^−/−^/Ras^V12^, indicating that Rb loss is unlikely to play any role in the CIN. The chromosome-destabilizing effect of HRas^V12^ was very rapid, and primary astrocytes were harvested for karyotyping in no more than a few cell cycles after infection (six days after infection). Although HRas^V12^ is able to induce numerical chromosomal instability by massive aneuploidy, it also causes structural chromosome abnormalities [23]. Hence, these numerical and structural chromosome abnormalities show that activated Ras is sufficient for inducing chromosomal instability in the absence of other signals, suggesting that Ras-induced chromosomal instability arises from ROS accumulation and DNA replication.

When we analyzed the karyotype of the cell lines T653 and T731, we found that of the cells analyzed in both cell lines, virtually 100% were aneuploid. Curiously, and in contrast to primary astrocytes, in the cell lines most of the cells analyzed showed a chromosome number greater than 60, and a large percentage of these presented >80 chromosomes per cell. These data suggest that in the process of tumorigenesis and in the successive passes suffered by the cell lines, there was a selection process that favored the cells with a higher number of chromosomes.

### Cytochrome c oxidase activity correlates with increased ROS generation

Since the intracellular accumulation of ROS is linked to increased mitochondrial activity, we analyzed the mitochondrial activity in our experimental groups. Mitochondrial activity can be measured by assaying for mitochondrial-specific enzymes. Specifically, we analyzed the mitochondria-specific cytochrome c oxidase activity in soluble and membrane-bound mitochondria samples. The enzyme cytochrome c oxidase is a large transmembrane protein located in the inner membrane of mitochondria, and it is the terminal electron acceptor in the electron transfer chain.

**Figure 3:**
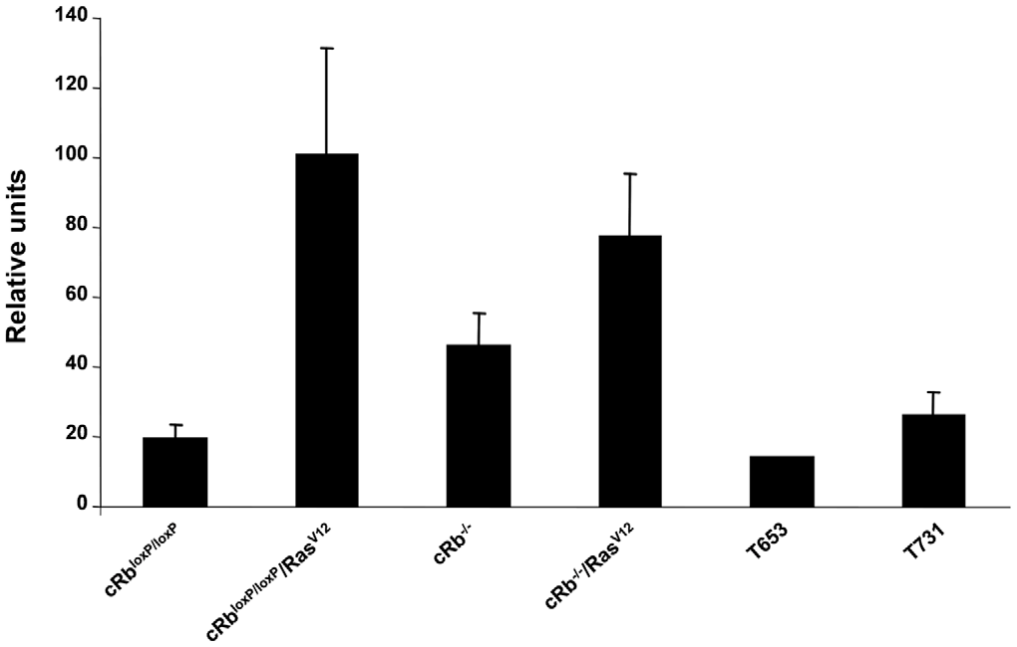
Quantification of cytochrome c oxidase activity. Comparison of respiratory activity between primary astrocytes (cRb^loxP/loxP^, cRb^−/−^, Rb^loxP/loxP^/Ras^V12^ and cRb^−/−^/Ras^V12^) and tumor cell lines revealed greater mitochondrial activity in astrocytes Rb^loxP/loxP^/Ras^V12^ and cRb^−/−^/Ras^V12^ than in the other cell groups, including cell lines T653 and T731.

When we determined cytochrome c oxidase activity in the different groups of cells, we observed that Rb^loxP/loxP^/Ras^V12^ and cRb^−/−^/Ras^V12^ astrocytes had a higher cytochrome c oxidase activity than cRb^loxP/loxP^, cRb^−/−^, T653 and T731 cells. In addition, this higher mitochondrial activity was correlated with the enhanced levels of ROS observed in Rb^loxP/loxP^/Ras^V12^ and cRb^−/−^/Ras^V12^ astrocytes (Figure 3).

Since the staining of astrocyte cells with DHE suggested that the mitochondria were the source of this ROS, we used MitoTracker Red CMXRos to study how oncogenic Ras affects the distribution and quantity of mitochondria. In order to label the mitochondria, the cells were simply incubated with MitoTrackerR probes, which passively diffuse across the plasma membrane and accumulate in active mitochondria, thus giving an estimate of the mitochondrial mass and position in cells.

**Figure 4:**
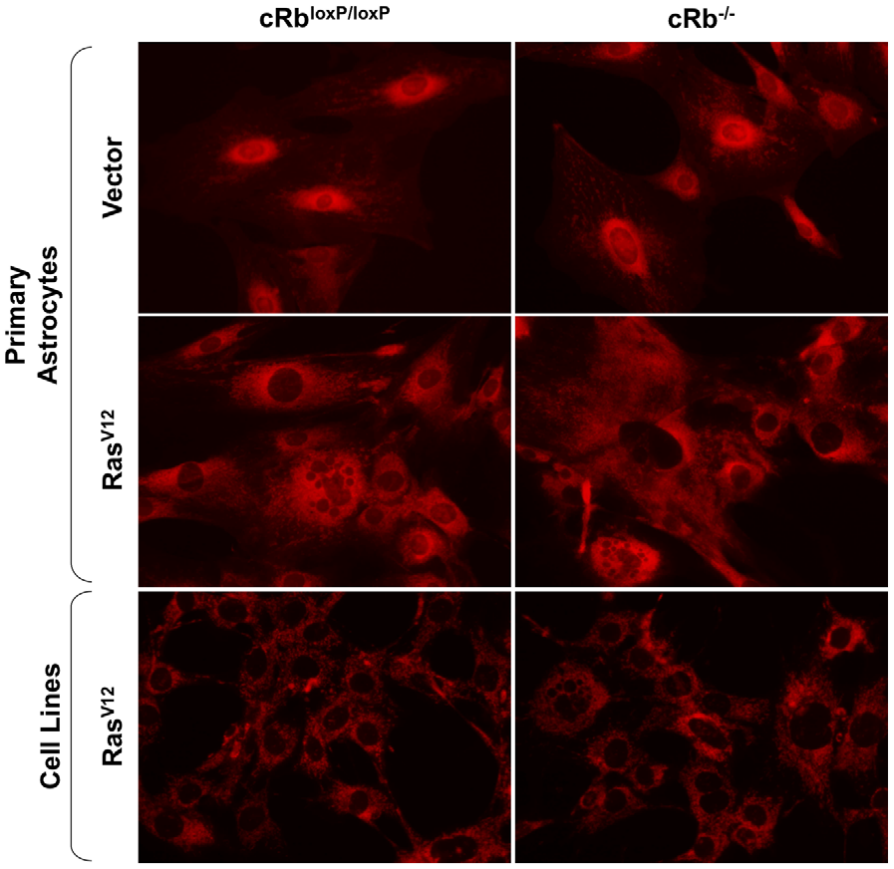
Mitochondrial changes in astrocytes. MitoTracker Red CMXRos fluorescence images of primary astrocytes 6 days after selection and astrocyte lines T653 and T731. Oncogenic *ras* expression induced distribution of the mitochondria throughout the cell cytoplasm, whereas control cells (Rb^loxP/loxP^ and cRb^−/−^) showed a concentration of mitochondria around the nuclei of cells.

After labeling with MitoTracker Red CMXRos, we observed red fluorescence all over the cytoplasm in cells expressing oncogenic Ras, showing an increase of mitochondrial mass in the primary astrocytes (Rb^loxP/loxP^/Ras^V12^ and cRb^−/−^/Ras^V12^) compared to the cell lines T653 and T731, in which the fluorescence was less intense. On other hand, we observed a dramatic increase in perinuclear mitochondrial mass in the control cells (cRb^loxP^/^loxP^) and astrocytes without Rb (cRb^−/−^), but we did not observe a decrease in the number of mitochondria per cell. The distribution of mitochondria around the nucleus implies a lower efficiency (Figure 4).

### Analysis of mouse mtDNA genomes

**Figure 5:**
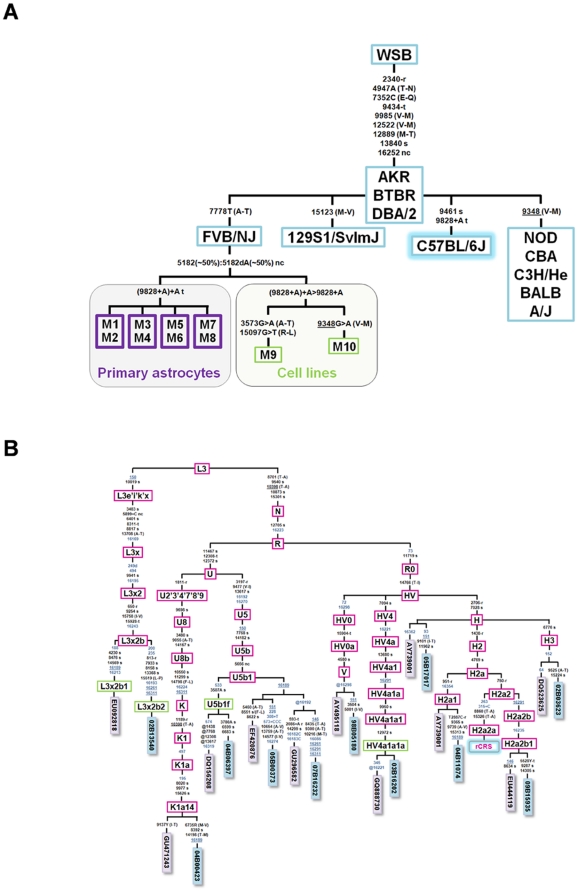
Maximum parsimony trees of mouse and human mtDNA sequences. (A) Maximum parsimony tree of mtDNA entire genomes of different mouse strains, primary cell cultures and cell lines. The phylogenetic skeleton of the different mouse strains was modified from Goios et al. (2007). The violet squares at the bottom refer to the FVB/NJ strain samples analyzed in the present study, whereas the green squares indicate the cell lines (sample ID is as in Table 1). Note that the C57BL/6J strain was used as the reference strain. Nucleotide changes along the branches are indicated with respect to the reference strain (C57BL/6J). All mutations are transitions unless a suffix specifies a transversion (A, C, G, T), an insertion (+), a deletion (d), a synonymous substitution (s), a mutational change in tRNA (-t), or amino acid replacements (indicated in round brackets). Recurrent mutational events are underlined. (B) Maximum parsimony tree of human mtDNA gliomas. The blue rectangles indicate the entire mtDNA genomes analyzed in the present study, whereas the violet rectangles are the genomes of healthy individuals phylogenetically to the gliomas analyzed in the present study (GenBank accession numbers are indicated). The mutations are displayed along the branches (mutations of the control region in blue script); the nomenclature is relative to the rCRS [24]. Haplogroups defined here for the first time are indicated in green rectangles. All mutations are transitions unless a suffix specifies a transversion (A, C, G, T), a deletion (d), an insertion (+), a synonymous substitution (s), a mutational change in tRNA (-t), a mutational change in rRNA (-r), or amino acid replacements (indicated in round brackets). Recurrent mutational events are underlined, whereas @ indicates back mutations. Several mutational hotspot variants were not considered for the phylogenetic reconstruction and therefore were eliminated from the tree; these included variants at the homopolymeric tracks around position 310, the microsatellite at m.523–524 (aka m.522–523), the transversions m.16182A>C, m.16183A>C, m.16193+1C(C) and m.16519T>C, and length or point heteroplasmies. The codes of the samples are indicated in blue rectangles at the terminal branches of the phylogeny. Note that sample DQ156208 [43] has an unusual number of reversions that are probably due to documentation or sequencing errors [44].

Primary mouse astrocytes, cRb^loxP/loxP^, cRb^−/−^, Rb^loxP/loxP^/Ras^V12^ and cRb^−/−^/Ras^V12^, as well as the two cell lines T653 and T731, were sequenced for their entire mtDNA genomes. In order to avoid false positives and/or negatives and documentation errors as much as possible [20], [21], we followed the forensic standards previously proposed by Cerezo et al. [22]. In agreement with the literature [31], all mouse samples differed from the reference sequence by homoplasmic transition 9461T>C and homoplasmic transversion 7778G>T [23] (Figure 5A). Moreover, all of the samples showed length heteroplasmy around the homopolymeric tract ranging from position 5172 to position 5182 (Figure 5A). The adenine homopolymeric tract located between positions 9821 and 9828 showed subtle length heteroplasmy in all astrocyte samples, but a more pronounced length heteroplasmy in the cell lines derived from the tumor samples (T653 and T731; Figure 5A). Slippage of the Taq polymerase could explain these heteroplasmies given the length of both homopolymeric tracks, namely 11 and 10 adenines, respectively. Instabilities at homopolymeric regions are normal in healthy individuals, as already demonstrated in human studies [32] (Figure 5A). Since all of these variants were ubiquitous in all of the samples, there is no reason to believe that they could play a role in the tumorigenic process. Finally, the cell line T653 also differed from the reference sequence by the heteroplasmies m.3573G/A and m.15907G/T (both positions at ∼60∶40), and cell line T731 by the heteroplasmy m.9348G/A (∼70∶30). The minor variants of the latter indicate that the heteroplasmies were non-synonymous, but this condition alone does not support a pathogenic role (Figure 5A). For instance, variant m.9348G>A defines a phylogenetic clade of mouse strains including NOD, CBA, C3H/He, BALB(2), and A/J [31].

### Analysis of human glioma mtDNA genomes

In addition, the mtDNA variation found in the entire genomes of 11 human glioma samples (Table S3) was compared to variations observed in human populations and reported in GenBank and the literature (>7800 entire genomes) [33] Most of this variation corresponded to expected polymorphisms according to the haplogroup adscription. Figure 5B shows the maximum parsimony tree of the 11 mtDNA genomes analyzed here and also, for the sake of comparison, the mtDNA phylogenetically related genomes observed in healthy individuals previously reported in GenBank and/or the literature. All of the samples belonged to typical European haplogroups, with the exception of sample #02B13540, which belonged to the sub-Saharan lineage L3x2b. The haplogroup distribution found in patient mtDNAs fitted well with the one expected in a sample of healthy individuals from the same geographical region [34]. Apart from the basal mutations, all of the mtDNA profiles carried their own set of private variants (tips of the phylogeny in Figure 5B) as was expected given the fact that not all human genetic variations have been recorded in the literature and databases. The gene locations and functional characteristics of all the variants observed in the human glioma mtDNAs are indicated in Table S4.

## Discussion

Maintenance of mitochondrial genome integrity is of critical importance to cell viability. In fact, the mechanisms that preserve this integrity are conserved through evolution. Respiration-deficient mutant yeast cells display elevated steady-state levels of ROS that are associated with impairment in their cellular antioxidant response, which compromises the viability of these cells and shortens their lifespan [35]. Moreover, mtDNA is susceptible to damage by an important number of anticancer agents, thus mtDNA is a critical target for several compounds currently used in the clinic, and a potential target for novel chemotherapeutic agents [36]. Altogether, these highlight the importance that the mitochondrial genome plays in cell survival both in normal and tumor cells.

In the present study, we used a glioma model as a paradigm of tumor initiation, progression and maintenance. As demonstrated here, when a normal cell becomes tumoral, the cell starts to respond to increased levels of ROS production with a rapid antioxidant response. This response gives the cell the capability of tolerating a toxic level of ROS; as a result, genomic instability occurs and facilitates oncogenic transformation of the cell. Once this transformation has taken place, the cell dramatically reduces both ROS generation and its antioxidant response. Some authors have described this so-called “mitochondrial silencing” as a decrease in the mitochondrial activity found in cancer cells, as reviewed in [37].

The mitochondrial genome was analyzed along the entire evolution of the transformation process. A total of eight mouse primary cell culture samples, two mouse cell line samples, and 11 human glioma samples were sequenced for the entire mtDNA genome. In order to avoid false claims of mtDNA instability [20], [21], a strict laboratory protocol was followed according to forensic standards [22]. In contrast to the high genomic instability observed at the nDNA level, no instability was observed in the mouse mtDNA complete genomes. On the other hand, the variations observed in the human glioma mtDNA were compared against the variation in the mtDNA genomes reported for healthy individuals in public databases and the literature (>7800 complete genomes). There is no *a priori* reason to believe that the variations observed in the human glioma mtDNA play a role in tumorigenesis because most of them are common and well-known polymorphisms in the worldwide of mtDNA phylogeny. The few new variants observed for the first time here fitted well with expectations given the fact that not all human variations are represented in databases. Our results are in good agreement with the hypothesis supported by [20], [21] from a theoretical basis, indicating that most of the reported instabilities in tumor studies are spurious, mainly due to the low quality DNA commonly used in such studies that leads to errors of a different nature [20], [21], [38].

In a mouse model of gliomagenesis, we showed that multiple and important alterations in the nDNA genome occur, as well as alterations in the metabolism of the mitochondria. In contrast with previous reports, the mtDNA molecule remains intact in tumorigenesis in both mice gliomagenesis and in human gliomas.

## Materials and Methods

### Cell lines, cell culture and retroviral transduction

Astrocytes were generated from c*Rb* ^loxP/loxP^ neonatal mice at day 3. The care and use of all experimental animals was in accordance with institutional guidelines and approved by the Ethics Committee of the University of Santiago de Compostela and Xunta de Galicia (approval ID 15005AE/07/FUN01/FIS02/JACP1). The cells were maintained in Dulbecco's modified Eagle medium (Sigma, St. Louis, MO, USA) with 10% fetal bovine serum and 1% L-glutamine (GIBCO-Invitrogen, Barcelona, Spain). In order to introduce an activated *Ras* allele and *Rb* loss into the astrocytes, *Phoenix*-Eco packaging cells (a gift from G.P. Nolan) were transfected with pBabe, pBabe-HRas^V12^, PIG-puro and PIG-CRE retroviral plasmids (a gift from P.P. Pandolfi). The T653 and T731 cell lines were generated from tumors derived from astrocytes modified by Rb^loxP/loxP^/Ras^V12^ and cRb^−/−^/Ras^V12^, respectively (Table 1).

### Determination of ROS production

Reactive oxygen species generation by astrocytes was detected with the fluorescent probe DHE (Dihydtroethidium; Molecular Probes, Carlsbad, CA, USA). Dihydtroethidium is a membrane-permeable compound, which is oxidized to red fluorescent ethidium (DNA binding membrane-impermeable compound) by the action of radicals (O_2_^−^). The astrocytes were incubated with 5 µM of DHE for 30 min at 37°C and protected from light. Finally, intracellular ROS production was determined by the increase in fluorescence observed by an Olympus IX70 fluorescence microscope under identical conditions in all experimental groups.

### Chromosome metaphase preparation

Metaphase spreads were prepared from exponentially growing cells after treatment with colcemid. Cells were incubated in a hypotonic buffer (0.05 M KCl, 0.0034 M trisodium citrate) for 20 min at 37°C and fixed in 75% methanol and 25% acetic acid. The cells were then spotted onto microscope slides and stained with 2% Wright in Gurr buffer (pH 7.0). Metaphase chromosomes were scored using a Leika 2005 microscope under a ×100 oil objective lens. T-FISH was performed on unstained metaphase chromosomes using a Cy3-labeled peptide nucleic acid probe. For T-FISH, both the DNA probe and the slides were heat denatured (80°C for 5 min) and hybridized at 37°C for 2 h, in accordance with the manufacturer's specifications (Dako Cytomation, Glostrup, Denmark). The slides were counterstained with DAPI and the images were captured using a Leika 2005 microscope equipped with the software program by Leika 4000. At least 50 metaphases were analyzed from three independent experiments.

### Mitochondrial characterization

The number of mitochondria and their distribution were determined using the MitoTracker Red CMXRos selective probe (Molecular Probes, Barcelona, Spain). The cells were incubated in a medium without FBS and containing 100 nM MitoTracker for 30 min at 37°C. The medium was then replaced with a complete medium but without MitoTracker. Microscopic examination with an Olympus IX70 fluorescence microscope was carried out.

### Electron transport chain enzyme activities

Fresh purified mitochondrial samples from primary astrocytes and cell lines in log-phase growth were used to monitor the mitochondrial respiratory function (MITOISO2; Sigma, St. Louis, MO, USA). Cytochrome c oxidase activity in membrane-bound mitochondrial samples was determined by measuring the absorption changes of cytochrome c at 550 nm. For this, we used the Cytochrome c Oxidase Assay Kit CYTOCOX1 from Sigma (St. Louis, MO, USA).

### DNA samples

Primary mouse astrocytes cRb^loxP/loxP^, cRb^−/−^, Rb^loxP/loxP^/Ras^V12^ and cRb^−/−^/Ras^V12^, as well as the two cell lines T653 and T731, were sequenced for the entire mtDNA genome.

The mtDNA genome of the strain C57BL/6J was used as the reference sequence [39]. The revised Cambridge Reference Sequence [40] was used as the reference for human samples.

### DNA extraction

The primary mouse astrocytes and cell line samples were solubilized with sodium dodecyl sulfate buffer and treated with proteinase k (1 h at 55°C), with subsequent precipitation of the DNA with ethanol. Human glioma samples were extracted following standard phenol-chloroform protocols.

### DNA sequencing of mouse and human mtDNA entire genomes

The protocols carried out for primer design, Polymerase Chain Reaction (PCR) and the sequencing reaction were previously described [34], although slight modifications were made in the present study. The mouse primers were designed in order to prevent them from annealing at varying positions among the strains. Table S1 indicates the sequence differences between the mtDNA genome of three different strains. Most of the primers were previously reported [41], [31]; some however, were modified in order to improve PCR efficiency (Table S2). Primer 3 software (http://frodo.wi.mit.edu/cgi-bin/primer3) was used to design the primers both PCR amplification and the sequencing reaction. All of them had an annealing temperature around 60°C and 58°C for the control regions in mouse and human DNA, respectively, and 55°C and 58°C for the coding regions in mouse and human DNA, respectively. The National Centre for Biotechnology Information (NCBI; http://www.ncbi.nlm.nih.gov) database was interrogated using BLAST in order to test the primers against possible repetitive sequences and sequence homologies in the nuclear genome. Each primer pair for PCR amplification and sequencing was selected independently. AutoDimer (http://www.cstl.nist.gov/biotech/strbase/AutoDimerHomepage/AutoDimerProgramHomepage.htm) was used to test for potential hairpin structures and primer-dimer problems. The melting temperature (Tm) was double-checked using Primer 3 and Oligo properties calculator (http://www.basic.northwestern.edu/biotools/oligocalc.html) (Table S2). Primers for the amplification and sequencing of human samples were previously described [42].

Polymerase chain reaction amplification was carried out in a 9700 Thermocycler (Applied Biosystems, Foster City, CA, USA) using the PCR master mix QIAGEN Kit Multiplex PCR (Qiagen, Dusseldorf, Germany). After a 95°C pre-incubation step for 15 min, PCR was performed in a total of 30–35 cycles using the following conditions: 94°C denaturation for 30 s, annealing at 55°C to 60°C (see details above) for 90 s, and extension at 72°C for 90 s, followed by a 10 min of final extension at 72°C and 12°C until removed from the thermocycler.

Before the sequencing reaction, the PCR products were purified using MultiScreen PCR μ 96 plates (Millipore, Billerica, Massachusetts, USA).

The sequencing reaction was carried out in a total volume of 10 µL with 0.5 µL of the BigDye® Terminator v3.1 Cycle Sequencing Kit (Applied Biosystems, Foster City, CA, USA), 2 µL of the 5× buffer of the BigDye kit, a primer at a concentration of 0.3 µM, plus 3.5–4.5 µL of the PCR-purified product. The reaction was performed in a 9700 Thermocycler (Applied Biosystems, Foster City, CA, USA) following the recommendations of the manufacturer: an activation step of 96°C for 3 min; then 25–30 cycles as follows: 96°C denaturation for 30 s, 50°C annealing for 15 s, and 60°C extension for 4 min. Unincorporated primers were eliminated by adding 1 µL of SAP (GE Healthcare Life Sciences, Little Chalfont, Buckinghamshire, United Kingdom) for 80 min at 37°C followed by 15 min at 85°C for enzyme inactivation. A final purification was carried out using the Montage™ SEQ_96_ Sequencing Reaction Cleanup Kit (Millipore, Billerica, Massachusetts, USA), following the manufacturer's instructions. Capillary electrophoresis was undertaken on an ABI PRISM 3730xl Genetic Analyzer (Applied Biosystems, Foster City, CA, USA). The resulting data were analyzed using Sequencing Analysis and SeqScape Software (Applied Biosystems, Foster City, CA, USA).

The six mouse and eleven human complete mtDNA genomes analyzed in the present study are available in GenBank under accession numbers HQ675026-HQ675031 and HQ675032-HQ675042, respectively. Note that four of the sequences of the primary astrocytes were replicated twice for the entire mtDNA genome but only recorded by a single GenBank accession number. The heteroplasmies and missing data (sample 04B11074) are indicated in GenBank records under the same IUPAC code: “N”

## Supporting Information

Table S1 Nucleotide differences between the mtDNA genomes of different mouse strains.
(DOC)

Table S2 Amplification and sequencing primers used for the mouse samples.
(DOC)

Table S3 Variants observed on the 11 human glioma mtDNAs sequenced in the present study for the entire genome, their gene locations and functional characteristics.
(XLS)

Table S4 Gene locations and functional characteristics of all the variants observed in the human glioma mtDNAs.
(XLS)
