# Supplementary material for: The Mitochondrial Genome Is a “Genetic Sanctuary” during the Oncogenic Process
Source: PLoS One. 2011 Aug 17;6(8):e23327. doi: 10.1371/journal.pone.0023327 (PMC3157371; doi:10.1371/journal.pone.0023327)
Supplement: Table S1 — Nucleotide differences between the mtDNA genomes of different mouse strains. (DOC) [file pone.0023327.s001.doc]

**Table S1**

| **Position** | **Mouse strains** | | | | | | | |
| --- | --- | --- | --- | --- | --- | --- | --- | --- |
|  | **C57BL/6J** | **FVB/NJ** | **129S1/SvImJ** | **NOD** | **CBA** | **A/J** | **C3H/He** | **BALB** |
| 7778 | G | T | G | G | G | G | G | G |
| 9461 | T | C | C | C | C | C | C | C |
| 9821-9828 | - | +A | +A | +A | +A | +AA | +TA | +A |
| 15123 | A | A | G | A | A | A | A | A |
| 9348 | G | G | G | A | A | A | A | A |
