# Supplementary material for: The Mitochondrial Genome Is a “Genetic Sanctuary” during the Oncogenic Process
Source: PLoS One. 2011 Aug 17;6(8):e23327. doi: 10.1371/journal.pone.0023327 (PMC3157371; doi:10.1371/journal.pone.0023327)
Supplement: Table S2 — Amplification and sequencing primers used for the mouse samples. (DOC) [file pone.0023327.s002.doc]

**Table S2**

| **Primer ID** | **Primer sequence** | **Position** | **Tm (Oligo Properties Calculator)** | **Design/References** |
| --- | --- | --- | --- | --- |
| m15300F | ataaacattactctggtcttgtaaacc | 15300-15326 | 62 | [1] |
| m094R | Attataaggccaggaccaaacct | 094-072 | 61 | [1]; modified |
| m15384F | Cagcacccaaagctggtatt | 15384-15403 | 58 | Present study |
| m040R | gcattttcagtgctttgctttg | 040-019 | 58 | Present study |
| m15818F | gcgttatcgcccatacgtt | 15818-15836 | 57 | Present study |
| m15881R | tgggctgattagacccgata | 15881-15860 | 58 | Present study |
| m16243F | TCATGTTCCGTGAACCAAAAC | 16243-16263 | 57 | Present study |
| m409R | tttaggtcctaacaatgaattttc | 432-409 | 57 | [2] |
| m348F | cttcggcgtaaaacgtgtcaac | 348-369 | 62 | [2] |
| m889R | gcggtgtgtgcgtacttcatt | 909-889 | 61 | [2] |
| m826F | aaactaaaggactaaggaggattt | 826-849 | 58 | [2] |
| m1368R | tttgccacatagacgagttgatt | 1390-1368 | 59 | [2] |
| m1307F | actaaaagaattacagctagaaac | 1307-1330 | 57 | [2] |
| m1754R | CGTATGCCTGGAGAATTGGA | 1773-1754 | 58 | Present study |
| m1607F | GCCTAAAAGCAGCCACCAATA | 1607-1627 | 60 | Present study |
| m2320R | atctgggtcaataagatatgttg | 2342-2320 | 58 | [2] |
| m2256F | tgacctcggagaataaaaaatcc | 2256-2278 | 59 | [2] |
| m2798R | ctactaatgttaggaaggctatg | 2820-2798 | 59 | [2] |
| m2763F | aatatcctaacactcctcgtcc | 2763-2784 | 60 | [2] |
| m3384R | CCGGCTGCGTATTCTACGTTA | 3403-3383 | 61 | Present study |
| m3275F | TCTGCCAGCCTGACCCATA | 3275-3293 | 60 | Present study |
| m3761R | ctattgtcctagaaataagaggg | 3783-3761 | 59 | [2] |
| m3685F | cgggagtaccaccatacatat | 3685-3705 | 60 | [2] |
| m4238R | taatcagaagtggaatggggc | 4258-4238 | 60 | [2] |
| m4178F | caaacaaacggtcttatccttaa | 4178-4200 | 58 | [2] |
| m4737R | ctatgagtgttgctataattagac | 4760-4737 | 58 | [2] |
| m4679F | ccactaacaggattcttaccaa | 4679-4700 | 58 | [2] |
| m4963R | TTGAAGGCTCGCGGACTAGTAT | 4984-4963 | 62 | Present study |
| m4861F | CCAAACAAAAACTAAACCCAACCT | 4861-4884 | 60 | Present study |
| m5225R | gtgatattcatgtcgaattgcaaa | 5248-5225 | 58 | [2] |
| m5183F | tggcggtagaagtcttagtag | 5183-5203 | 60 | [2] |
| m5727R | cctgcatgggctagatttcc | 5746-5727 | 60 | [2] |
| m5655F | ctccttctcctagcatcatcaa | 5655-5676 | 60 | [2] |
| m6206R | tgtctacatctaatcctactgtg | 6228-6206 | 59 | [2] |
| m6141F | ggaatagtatgagcaataatgtc | 6141-6163 | 58 | [2] |
| m6569R | ACTCCTACGAATATGATGGCG | 6589-6569 | 60 | Present study |
| m6409F | CATCCCTTGACATCGTGCTT | 6409-6428 | 58 | Present study |
| m7179R | ggtttcaacttcttgtgcatcta | 7201-7179 | 59 | Present study |
| m7119F | ccttagtcctctatatcatctc | 7119-7140 | 58 | Present study |
| m7642R | tattttagtggaaccatttctagg | 7665-7642 | 58 | [2] |
| m7581F | ggttattctatggccaatgctc | 7581-7602 | 60 | [2] |
| m8119R | ATTATTAGGGTTCATGTTCGTCC | 8141-8119 | 59 | [2] |
| m8041F | AACAACCGTCTCCATTCTTTC | 8041-8061 | 57 | [2] |
| m8593R | gtgggtcattatgtattatcatg | 8615-8593 | 58 | [2] |
| m8553F | tcaagcctacgtattcaccct | 8553-8573 | 60 | [2] |
| m9063R | tttatgtggtttcgtttaccttc | 9085-9063 | 58 | [2] |
| m9011F | agtacttctagcatcaggtgttt | 9011-9033 | 59 | [2] |
| m9572R | TTGTAGGGTCGAATCCGCAT | 9591-9572 | 58 | [2]; modified |
| m9519F | GCATTCTGACTCCCCCAAATAA | 9519-9540 | 60 | [2] |
| m10119R | atctgttccgtacgtgtttgaa | 10140-10119 | 58 | [2] |
| m10060F | accatcttagttttcgcagcc | 10060-10080 | 60 | [2] |
| m10613R | gaaccgattagggtataaaatagg | 10636-10613 | 60 | [2] |
| m10570F | gatgagggaaccaaactgaac | 10570-10590 | 60 | [2] |
| m11095R | CGATTATTAGTATTGTTGCTCCTA | 11118-11095 | 58 | [2]; modified |
| m11016F | TCACTAATCGCCTACTCCTCAGTT | 11016-11039 | 64 | [2]; modified |
| m11563R | gtcagattcacagtctaatgttt | 11585-11563 | 58 | [2] |
| m11495F | acttattcttctaactaccagtc | 11495-11517 | 58 | [2] |
| m12066R | gattgatgtttgggtctgagtg | 12087-12066 | 60 | [2] |
| m12018F | gccctttttgtcacatgatcaat | 12018-12040 | 59 | [2] |
| m12566R | GGCTCCGAGGCAAAGTATAGTT | 12587-12566 | 62 | [2]; modified |
| m12511F | TTTTCCTACTGGTCCGATTCCA | 12511-12532 | 60 | [2] |
| m13068R | aggtctgggtcattttcgttaat | 13090-13068 | 59 | [2] |
| m13008F | agcatacgaatcatttacttcgt | 13008-13030 | 58 | [2] |
| m13567R | gcgggtatttttattattatcgag | 13590-13567 | 58 | [2] |
| m13499F | gtactttatatcattcctaattaac | 13499-13523 | 56 | [2] |
| m14016R | TTTTTGGTTGGTTGTCTTGGGT | 14037-14016 | 58 | [2] |
| m13963F | CCCACTAACAATTAAACCTAAACCT | 13963-13987 | 61 | [2]; modified |
| m14509_R | ataaatgctgtggctatgactg | 14530-14509 | 58 | [2] |
| m14432F | tcatgtcggacgaggcttata | 14432-14452 | 60 | [2] |
| m14980R | ggggattgagcgtagaatgg | 14999-14980 | 60 | [2] |
| m14921F | taatccactaaacaccccacc | 14921-14941 | 60 | [2] |
| m15484R | AATGTACTAGCTTATATGCTTGGG | 15507-15484 | 60 | [2]; modified |

Primers are referred to the 5’ position and regarding C57BL6J
